# Reducing the frequency of hand hygiene to maintain skin integrity among nurses in the growing care unit: A pilot study

**DOI:** 10.20407/fmj.2024-029

**Published:** 2025-04-17

**Authors:** Masami Tano, Masushi Kohta, Yoshiko Yano, Junko Sugama

**Affiliations:** 1 Department of Nursing, Fujita Health University Hospital, Toyoake, Aichi, Japan; 2 Research Center for Implementation Nursing Science Initiative, Fujita Health University, Toyoake, Aichi, Japan

**Keywords:** Hand hygiene, Infection prevention, Skin physiology

## Abstract

**Objectives:**

Consistent hand hygiene practice is required to reduce the prevalence of healthcare-associated infections. However, frequent hand hygiene compromises the skin barrier, causing hand eczema. Consequently, compliance with this practice can be reduced. This study aimed to determine the safety of reducing the frequency of hand hygiene by nurses, focusing on the de-implementation of the current excessive hand hygiene protocol.

**Methods:**

A single-group, pretest–post-test pilot study was conducted in three nurses at a growing care unit (level 2 neonatal intensive care unit) in a Japanese university hospital. The developed intervention was performed four times and the current hand hygiene protocol was performed six times in each nurse. The number of microbial contaminations on the hands at each time point was the primary outcome. Impairment of the skin barrier (changes in the stratum corneum water content, transepidermal water loss, and skin pH) was the secondary outcome.

**Results:**

The pre- and post-test residual bacterial contamination at each time point was not significantly different (*p*=0.99). The amount of change in skin physiology was also not significantly different between the pre- and post-tests.

**Conclusions:**

Hand hygiene effects, such as a reduction in bacterial contamination and impairment of skin physiology, were significantly different between before and after the intervention of reducing the frequency of hand hygiene by nurses. To confirm this finding, we will focus on resistant bacteria and test this intervention in randomized, controlled trials.

## Introduction

Preventing healthcare-associated infections in growing care units (GCUs, level 2 neonatal intensive care units providing convalescent care after intensive care) is crucial in hospitals. Neonates admitted to the GCU are predisposed to hospital-acquired infections because of their underdeveloped immune systems, the requirement for frequent invasive procedures, and generally longer hospitalization.^1,2^ Healthcare-associated infections in neonates are associated with increased healthcare costs and a prolonged hospital stay, as well as multiple organ injuries and mortality.^3–5^

Hand hygiene, which is the practice of decontaminating the hands by washing with soap and water or using alcohol hand rub, is an elementary method for breaking the chain of infection.^6^ In fact, healthcare workers are the major vectors of multidrug-resistant organisms.^7^ Furthermore, direct patient contact is a major risk factor for bacterial contamination and subsequent transmission in healthcare workers.^8^ Skin contact, respiratory care, and diaper changes by healthcare workers during neonatal care are independently associated with an increased bacterial count in healthcare workers.^9^ Nurses usually have the most frequent patient–care interactions. Therefore, nurses have more opportunities than other hospital staff to practice hand hygiene.^10,11^

The development of hand eczema is a considerable barrier to improving the compliance of hand hygiene.^12^ The prevalence of self-reported hand eczema in nurses was 55%, 18%, and 35% in the US, Germany, and Japan, respectively.^13–15^ Affected nurses often complain of dryness or a burning sensation, rough and erythematous skin, and scaling or fissures. Cumulative exposure to irritants from hand hygiene products directly damages the skin surface, initiating a cascade of inflammatory changes.^16^ Recently, nurses have become highly at risk for hand eczema because of the increased requirement of handwashing and disinfection during the coronavirus disease 2019 pandemic.^17^ Skin damage alters the cutaneous bacterial flora, facilitating more frequent colonization of staphylococci and gram-negative species.^18^ There is a trade-off between increasing frequency of hand hygiene and reducing the incidence of hand eczema. Unfortunately, definitive methods of maintaining the balance between them remain unavailable.

In recent years, issues pertaining broadly to reducing (frequency or intensity) or stopping the use of harmful, ineffective, low-value, or unproven health services and practices have become more salient.^19^ De-implementation might improve outcomes and decrease healthcare costs.^20^ Most studies that evaluated interventions for hand hygiene practice aimed to improve adherence to guidelines. However, no studies have reported on reducing the frequency of hand hygiene to achieve the de-implementation of the current excessive hand hygiene protocol. Consequently, hand eczema has become increasingly prevalent. Therefore, this study aimed to determine the safety of reducing the frequency of hand hygiene by GCU nurses using a new hand hygiene protocol.

## Methods

### Study design

This pilot study aimed to develop a new hand hygiene protocol. Therefore, we examined the potential effects of reducing the frequency of hand hygiene using a single group, pretest–post-test study design. This study, which was conducted from August 2022 to March 2023, was approved by the Institutional Review Board of Fujita Health University (approval number: HM22-027) and registered (UMIN 000050259) by the University Hospital Medical Information Network Clinical Trials Registry in Japan. This study conformed to the principles of the Declaration of Helsinki, and participants’ data were kept confidential.

### Hypothesis

We hypothesized that residual bacterial contamination on the hands at each time point would not be significantly different between the developed intervention and the current hand hygiene protocol.

### Inclusion and exclusion criteria

Nurses working at the GCU in our university hospital and providing direct patient care that requires frequent hand hygiene were included in this study. However, those with any skin injury (e.g., cuts and wounds), except for hand eczema, on their hands were excluded.

### Procedure

We displayed a poster in the GCU to recruit participants. The poster described the purpose of the study, study outline, and required time to conduct the testing. To avoid coercion or undue influence, none of the researchers made direct contact with potential participants, in accordance with our university’s internal ethics rule. Nurses who wished to participate telephoned or e-mailed the researcher and further received a face-to-face explanation of the study purpose and requirements for participation. All participants provided written informed consent before being included in this study. The participation was entirely voluntary, and no incentives were offered.

The sample size was planned to be small because we intended to perform a pilot study, with only three participants in this study protocol. This sample size represents approximately 10% of the number of nurses working in the GCU.

### Intervention

**Figure 1:**
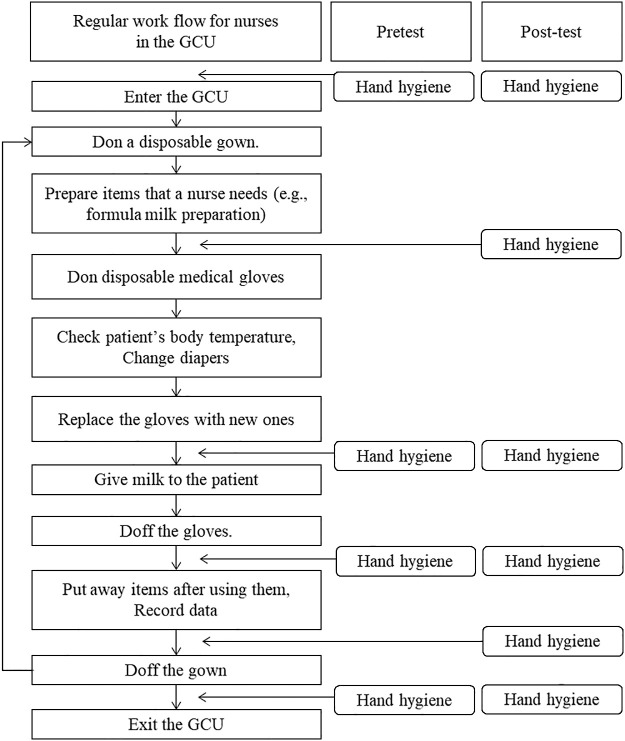
The hand hygiene technique was compared between the developed intervention (4 times) and the current protocol (6 times) during a regular workflow of participants in the day time for 2 consecutive days. When working with multiple patients in a day, the participants repeated the workflow from donning a disposable gown to removing the gown. GCU, growing care unit.

The developed intervention and the current hand hygiene protocol were performed four and six times in each nurse, respectively. The difference between the two protocols was whether hand hygiene was performed before donning disposable medical gloves and before removing gowns (Figure 1). Although the frequency of hand hygiene has not been standardized at the international level, hand hygiene is performed six times by the hospital’s infection control team in the GCU. Consequently, the nurses’ skin conditions have worsened, which has resulted in them lowering their adherence to this protocol. We discussed the reduction in the frequency of hand hygiene with the chief manager of nursing and the nursing staff. First, they proposed to stop the hand hygiene practice before donning disposable medical gloves. To reduce the occurrence of infection and complications, environmental cleaning procedures with an approved disinfectant has been performed twice daily in the patient care areas in the GCU. In addition, hand hygiene has been strictly conducted for healthcare workers and visitors when entering the GCU. As a result, the risk of bacteria being transferred to the hands of nursing staff was minimized, even if hand hygiene practice was not conducted before donning disposable medical gloves. Stopping hand hygiene practice before removing gowns was then proposed. The environmental cleaning procedures described above were expected to contribute to a reduction in bacterial contamination of computers for recording data and any items for patient care. Nurses usually have no opportunity to be in contact with patients between completing data recording and exiting the GCU. Therefore, the chief manager of nursing and the nursing staff decided to reduce the frequency of performing hand hygiene and change the timing of hand hygiene, thus leading to a new protocol (called the “intervention” in this study).

### Outcome measurement

We evaluated each participant for 18 days and measured the outcomes before and after the intervention. We conducted a pre-test on days 1 and 2 of the first week of the study, and a post-test on days 3 and 4 of the third week of the study; therefore, the time interval was 14 days. The current hand hygiene protocol was used on the first 16 days of the pre-test and during the interval, whereas the developed intervention was performed on the 2 remaining days of the post-test.

In the pre-test and post-test, measurements were performed for 2 days consecutively during the daytime shift. Before the participants started working, their skin physiology parameters at baseline were measured at a laboratory at approximately 08:00 h by a researcher who was adequately trained to operate equipment for such measurements. Bacterial contamination of the hands was assessed eight times daily. After a full working day (approximately 17:00 h), the participants visited the laboratory again, and the same measurements were taken as those taken in the morning.

### Primary outcome

The primary outcome was the result of comparisons of residual microbial contamination on the hands between the pre-test and post-test, as assessed by changes in the number of bacterial colony-forming units (CFUs). When collecting microbiological samples, each participant’s right hand directly touched a hand-shaped Trypticase Soy Agar medium plate (Eiken Chemical Co., Ltd., Tokyo, Japan) to measure bacterial contamination. These samples were collected at eight time points each day as follows: 1, before entering the GCU; 2, immediately after entering the unit; 3, before donning disposable medical gloves; 4, after replacing the gloves; 5, after removing the gloves; 6 after data entry; 7, after removing the gown; and 8, immediately after exiting the unit (Figure 1). After 48 h of incubation aerobically at 37°C, we counted the total number of CFUs in each plate.

### Secondary outcome

The secondary outcome was impairment of the skin barrier according to changes in water content of the stratum corneum, transepidermal water loss (TEWL), and the skin’s pH in both groups. Before and after each working day, these changes were measured at three points (thenar region, palm, and tip of the thumb) on each participant’s left hand. Measurements were made after resting for at least 15 min in a laboratory under controlled conditions of ambient temperature (22°C±1°C) and humidity (50%±10% relative humidity) to acclimatize themselves to laboratory conditions. We used a Corneometer CM825, Tewameter TM300, and skin pH meter PH 905 (all from Courage+Khazaka Electronic Gmbh, Köln, Germany) to measure the water content of the stratum corneum, TEWL, and skin pH, respectively. These devices were connected to a Multi Probe Adapter (Courage+Khazaka Electronic Gmbh), and all parameters were measured three times in each participant.

### Variables

Hand hygiene techniques using soap and alcohol-based hand rubs were not significantly different between the developed intervention and the current protocol.^21^ The same soap (Prime Wash; Saraya Co., Ltd., Osaka Japan) and a hand sanitizer (RABIGEL®; KENEI Pharmaceutical Co., Ltd., Osaka, Japan) were applied throughout the study period.

Nurses are often responsible for multiple patients each day. However, when planning this study, knowing the number of patients they had to care for each day during the study period was difficult. In addition, taking measurements during care for every patient was impractical because of the participants’ busy schedules. Therefore, the participants arbitrarily selected one patient to be measured each day.

### Statistical analysis

All data are expressed as the mean and standard deviation. In microbial evaluation, statistical significance between the two groups was evaluated using a two-way analysis of variance. The reduction rate of CFUs in each plate at each time point compared with baseline was calculated as the mean value and 95% confidence interval. Regarding skin physiology, changes in skin barrier function parameters before and after each working day were compared between the two groups, using a two-way analysis of variance. A *p* value <0.05 was considered statistically significant. All statistical data were analyzed using the Statistical Package for the Social Sciences version 28.0 (IBM Corporation, Armonk, NY, USA).

## Results

### Participants’ characteristics

The mean age of the three participants was 36±9.2 years, and all of them were women, with 13±8.2 years of work experience as nurses.

### Primary outcome

**Figure 2:**
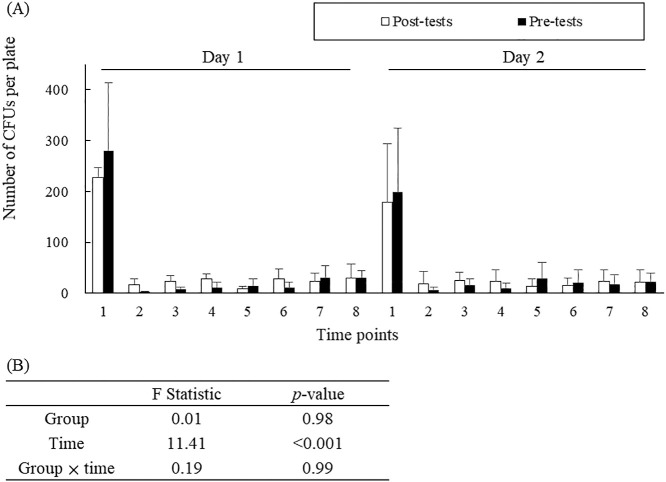
Results of bacterial contamination in the pre- and post-tests according to performing hand hygiene for 2 consecutive days. (A) The total number of CFUs in each plate was counted after 48 h of aerobic incubation. Time points: 1, before entering the growing care unit; 2, immediately after entering the growing care unit; 3, before donning disposable medical gloves; 4, after replacing the gloves; 5, after removing the gloves; 6, after recording data; 7, after removing the gown; and 8, immediately after exiting the growing care unit. (B) The two-way analysis of variance based on the group and timing showed no significant impairment. CFU, colony-forming unit.

Figure 2 shows that the rate of bacterial contamination at each time point each day was not significantly different between the pre- and post-tests (F=0.19, *p*=0.99). In the intervention, the mean numbers of CFUs at time point 1 on days 1 and 2 were 227±20.0 and 178±80.4, whereas those at time point 2 on days 1 and 2 were significantly reduced to 16±10.1 and 19±10.3, respectively. These numbers slightly increased from time point 2 to time points 3–8. The percentage of reduction in bacterial contamination relative to time point 1 ranged from 86.9% to 98.9%, except for that at time points 7 and 8 on day 1 in the pre-test (Table 1).

### Secondary outcomes

In the pre- and post-tests, the water content of the stratum corneum after working was lower than that before working each day (Table 2). The skin tended to restore this water content as shown by the finding that the hydration values before working on day 2 were higher than those after exiting the ward on day 1. The stratum corneum water content showed no significant impairment in either test at each measurement point (Table 2). The values of TEWL and skin pH were stable throughout all measurements, with no significant differences in the pre- and post-tests for each measurement point (Tables 3 and 4).

## Discussion

Currently, all healthcare workers should take more infection control measures because of the global spread of coronavirus disease 2019. Hand hygiene leads to a reduction in the number of transient bacteria on people’s hands. However, frequent and excessive hand hygiene compromises the skin barrier, leading to persistent inflammation and tissue damage. Therefore, healthcare workers tend to be less compliant with the hand hygiene practice. We aimed to determine how to de-implement the current frequent hand hygiene protocol. As the first step, we conducted a pilot study to determine whether reducing the frequency of hand hygiene practice on a workday is safe among Japanese GCU nurses, who are required to perform strict infection control measures in their daily practice.

Our study showed that many pathogens were present on the participants’ hands before entering the GCU, but they were considerably removed by hand hygiene. At work, the rate of bacterial contamination remained low. The effect of the reduction in bacterial contamination was not significantly different between the pre- and post-tests (Figure 2). Therefore, our developed intervention (reduced frequency of hand hygiene) may only have a slight effect on the risk of bacterial transmission from the hands of nurses.

Furthermore, we evaluated the effect of performing hand hygiene on the skin’s barrier function according to three parameters, namely, the stratum corneum water content, TEWL, and skin pH, as the secondary outcomes. The extent of impairment of the skin’s barrier function was not significantly different between the pre- and post-tests (Tables 2, 3, and 4). The difference in the frequency of performing hand hygiene may have had a slight effect on impairing skin physiology because the measurements were taken over a short period of 2 work days. A study with a longer follow-up duration might better determine the relationship between the frequency of performing hand hygiene and hand physiology.

Bacterial contamination of the hands results from exposure to the hospital environment and poor hygiene of healthcare workers, visitors (e.g., the patient’s parents), or even the patients themselves. The most serious issue facing infection control in the GCUs is the transient presence of *Staphylococcus aureus* flora, which can be methicillin-resistant. Approximately 22.9% of infants were reported to be colonized by *S. aureus* at some point during their hospital stay.^22^ Very preterm and very-low-birth-weight infants have an increased risk of methicillin-resistant *S. aureus* (MRSA) colonization.^23^ The prevalence of methicillin-sensitive *S. aureus* and MRSA in healthy newborns is 10.0% and 0.5%, respectively.^24^ Therefore, in the future, we will focus on MRSA among all pathogens and further investigate whether our developed hand hygiene procedure reduces the rate of MRSA transmission from patients to nurses (and vice versa) in randomized, controlled trials.

This study has two limitations. First, the one-group, pretest–post-test study design had a possibility of bias on the outcome reporting because the experimental and control groups were not randomly assigned. Nevertheless, this pilot study mainly aimed to determine the safety of reducing the frequency of hand hygiene by nurses working at the GCU. Our objectives were fully achieved, and the effects of a reduction in residual bacterial colonization and maintenance of skin physiology were similar between the pre- and post-tests. Second, the effect sizes to express the strength of the effects were relatively unstable and small. The upper limit of the 95% confidence intervals crossed 100%, suggesting that the confidence intervals shown in Table 1 were too wide because of the smaller numbers in the analysis. Further research involving a large sample size is necessary to determine the effect evaluation.

## Conclusions

This pilot study showed no significant differences in hand hygiene effects, such as a reduction in bacterial contamination and impairment of skin physiology, before and after the intervention of reducing the frequency of hand hygiene by nurses. To verify this result, we will focus on resistant bacteria among all pathogens and further test this intervention in a randomized, controlled trial with an adequate sample size.

## Figures and Tables

**Table 1:** Percentage of reduction of bacterial colonization in the pre- and post-tests at each time point relative to time point 1

Time point		Post-tests		Pre-tests	
		Mean	95% CI	Mean	95% CI
Day 1	1	Reference		Reference	
	2	92.7%	86.25–99.15	98.9%	98.00–99.81
	3	89.6%	83.82–95.37	94.9%	86.98–102.82
	4	88.3%	83.32–93.28	95.7%	92.76–98.64
	5	96.3%	93.01–99.58	94.0%	89.02–98.97
	6	88.2%	77.22–99.17	96.7%	94.01–99.30
	7	89.8%	80.86–98.74	78.0%	41.22–114.78
	8	86.9%	71.62–102.17	81.8%	58.03–105.56
Day 2	1	Reference		Reference	
	2	93.1%	84.83–101.36	97.8%	95.98–99.61
	3	87.6%	75.26–99.94	93.5%	89.20–97.80
	4	90.6%	82.22–98.97	96.5%	93.44–99.55
	5	95.0%	89.56–100.43	90.4%	80.32–100.47
	6	93.7%	89.28–98.11	93.2%	85.27–101.12
	7	90.5%	83.48–97.51	94.3%	87.85–100.75
	8	91.5%	83.33–99.64	90.2%	85.78–94.61

CI, confidence interval.

**Table 2:** Results of the change in the stratum corneum water content by hand hygiene before and after working

Mp.	Group	Day 1		Day 2		Source	F	*p*
		T0 Mean (SD)	T1 Mean (SD)	T0 Mean (SD)	T1 Mean (SD)			
Thenar region	Post.	47 (16.5)	40 (11.8)	40.1 (11.8)	38 (11.4)	G	0.03	0.86
	Pre.	51 (5.2)	39 (9.2)	45 (17.6)	39 (18.7)	T	0.14	0.94
						G×T	0.01	0.99
Palm	Post.	39 (9.8)	36 (3.7)	37 (4.3)	34 (5.3)	G	0.40	0.53
	Pre.	55 (7.7)	40 (6.9)	46 (0.7)	36 (8.2)	T	0.18	0.91
						G×T	0.07	0.97
Tip of the thumb	Post.	44 (9.5)	40 (9.7)	43 (6.1)	38 (9.2)	G	0.00	0.95
	Pre.	49 (8.5)	39 (6.5)	41 (4.6)	39 (8.8)	T	0.08	0.97
						G×T	0.02	0.99

Mp., measurement point; F, F statistic; T0, before the working day; T1, after the working day; SD, standard deviation; Post., post-test; Pre., pre-test; G, group; T, time.

**Table 3:** Results of the change in transepidermal water loss by hand hygiene before and after working

Mp.	Group	Day 1		Day 2		Source	F	*p*
		T0 Mean (SD)	T1 Mean (SD)	T0 Mean (SD)	T1 Mean (SD)			
Thenar region	Post.	56 (10.3)	57 (10.6)	56 (5.9)	54 (11.5)	G	0.01	0.95
	Pre.	62 (4.4)	63 (3.5)	57 (2.6)	54 (6.9)	T	0.01	0.99
						G×T	0.03	0.99
Palm	Post.	47 (9.5)	50 (12.9)	5.6 (0.3)	5.6 (0.2)	G	0.02	0.88
	Pre.	54 (7.5)	54 (9.9)	5.8 (0.2)	5.9 (0.1)	T	0.03	0.99
						G×T	0.02	0.99
Tip of the thumb	Post.	66 (15.0)	68 (19.1)	67 (7.2)	66 (8.0)	G	0.09	0.76
	Pre.	74 (9.9)	81 (13.4)	71 (14.1)	67 (5.7)	T	0.03	0.99
						G×T	0.01	0.99

Mp., measurement point; F, F statistic; T0, before the working day; T1, after the working day; SD, standard deviation; Post., post-test; Pre., pre-test; G, group; T, time.

**Table 4:** Results of the change in skin pH by hand hygiene before and after working

Mp.	Group	Day 1		Day 2		Source	F	*p*
		T0 Mean (SD)	T1 Mean (SD)	T0 Mean (SD)	T1 Mean (SD)			
Thenar region	Post.	5.6 (0.4)	5.5 (0.3)	5.4 (0.4)	5.2 (0.2)	G	0.09	0.77
	Pre.	5.8 (0.2)	5.9 (0.1)	6.3 (0.1)	6.1 (0.3)	T	0.01	1.00
						G×T	0.01	1.00
Palm	Post.	5.6 (0.3)	5.6 (0.2)	5.5 (0.2)	5.2 (0.4)	G	0.09	0.76
	Pre.	5.8 (0.2)	5.9 (0.1)	6.2 (0.3)	5.9 (0.1)	T	0.00	1.00
						G×T	0.01	1.00
Tip of the thumb	Post.	5.3 (0.3)	5.5 (0.2)	5.8 (0.2)	5.4 (0.5)	G	0.03	0.86
	Pre.	5.6 (0.6)	5.7 (0.3)	5.9 (0.2)	5.9 (0.1)	T	0.01	1.00
						G×T	0.00	1.00

Mp., measurement point; F, F statistic; T0, before the working day; T1, after the working day; SD, standard deviation; Post., post-test; Pre., pre-test; G, group; T, time.
